# Examining the Roles of Parent–Child Gender Dyads in the Association Between Parental Psychological Control and Adolescent Depressive Symptoms in Chinese Families

**DOI:** 10.3390/bs16040605

**Published:** 2026-04-19

**Authors:** Yuan Zhang, Shanhong Luo, Linda C. Halgunseth, Erin A. Moeser-Whittle, Anthony Hubert, Mary A. Balogun, Hao Wu

**Affiliations:** 1 T. Denny Sanford School of Social and Family Dynamics, Arizona State University, Tempe, AZ 85287, USA; yzha1325@asu.edu; 2 Department of Psychology, Fayetteville State University, Fayetteville, NC 28301, USA; sluo@uncfsu.edu (S.L.); emoeserw@uncfsu.edu (E.A.M.-W.); ahubert@uncfsu.edu (A.H.); mbalogun1@broncos.uncfsu.edu (M.A.B.); 3 Human Development and Family Studies, Michigan State University, East Lansing, MI 48824, USA; 4 Department of Psychology, Guangzhou Medical University, Guangzhou 511436, China; 2016990010@gzhmu.edu.cn

**Keywords:** gender dyads, parental psychological control, depressive symptoms, Chinese adolescents, Chinese fathers

## Abstract

Although Chinese parents’ use of psychological control has been linked with adolescent mental health, no studies to our knowledge have considered how the association may differ across gender dyads of parents and adolescents and minimal research has examined the joint influences of Chinese mothers’ and fathers’ use of psychological control on adolescent depressive symptoms. Participants included 3069 Chinese adolescents who rated their depressive symptoms as well as their mothers’ and fathers’ use of psychological control. Regression results revealed that the positive association between fathers’ psychological control and depressive symptoms was significant at low but not at high levels of mothers’ psychological control. Moreover, the positive association between fathers’ psychological control and depressive symptoms was significant for daughters but not for sons. Mothers’ psychological control was consistently positively associated with adolescent depressive symptoms. Findings from this study provide a more nuanced understanding of how gender dyads within Chinese families may influence the link between parental psychological control and adolescent depressive symptoms and highlight the importance for mental health programs to include fathers in their treatment plans when working with Chinese adolescents and their families.

## 1. Introduction

In China, 24.6% of adolescents are diagnosed with depression with girls reporting greater depressive symptoms than boys (X. Fu et al., 2021). Parental psychological control has been reported to be associated with greater adolescent depressive symptoms in the U.S. and China (e.g., Barber, 1996; Barber & Harmon, 2002). However, most studies on parental psychological control have not considered the joint effects of mothers’ and fathers’ control on adolescent well-being (Bi et al., 2024; Luebbe et al., 2018; Nigela et al., 2015), and no known study has examined whether this association differs across gender dyads (i.e., mother–daughter; mother–son; father–daughter, father–son) in Chinese families. Additional consideration of gender is needed to gain a more nuanced understanding of parent–child dynamics in Chinese families and their association with adolescent mental health, particularly for girls, who are at great risk of depressive symptoms (X. Fu et al., 2021; Yang et al., 2025; Yu et al., 2021).

Parental psychological control (e.g., love withdrawal, guilt induction, shaming) has been described as intrusive, manipulative, and rejecting parental behaviors that are linked to aversive developmental results in adolescents’ self-development and psychological well-being (Barber, 1996; Barber et al., 2005; Yu et al., 2021). In China, a few studies have found a negative association between Chinese parents’ psychological control and children’s mental health problems (Leung & Shek, 2020), external/internal problem behaviors (Barber, 1996), and social adjustment barriers (Q. Li et al., 2024). For example, Luebbe et al. (2018) found in a sample of 247 highschoolers and their parents (60% mothers) in China that psychological control mediated the positive association between parents’ strong academic achievement goals and adolescents’ anxiety. Similarly, studies based in the U.S. have shown that when adolescents perceive their parents as controlling, they are more likely to report an increase in depressive symptoms (Barber et al., 2005; Chyung et al., 2022; Tang et al., 2024; Zhu et al., 2023).

To understand the associations between parental psychological control and children’s depressive symptoms during adolescence—a developmental period in which depressive symptoms increase most rapidly, especially among females (Kwong et al., 2019; Shorey et al., 2022; World Health Organization, 2025)—we first consider Interpersonal Parental Acceptance–Rejection Theory (IPARTheory; Rohner, 2021). In IPARTheory, parental warmth is conceptualized as a continuum with parental acceptance and parental rejection at opposite ends (Rohner, 2021). Several studies have considered psychological control as a form of parental rejection because (1) it consists of rejection behaviors such as love withdrawal and (2) when parents exert psychological control, adolescents feel that their autonomy, individuality, and sense of self are being rejected (Nelson et al., 2013; Rohner, 2021; Zhu et al., 2023). According to IPARTheory, perceived parental acceptance is required for positive psychological adjustment in adolescence, while parental rejection is associated with negative psychological adjustment (Rohner, 2021).

Although this study is based on IPARTheory, it is also guided by an ecological perspective (Bronfenbrenner, 1986), which posits that sociocultural and familial contexts should be considered when seeking to understand adolescent development. Although not a monolith, many Chinese families may continue to endorse traditional values and practices. For example, traditional Chinese parenting is described as heavily influenced by the two central Chinese value systems—Confucianism and Taoism (Y. Li et al., 2010; Peterson et al., 2005). Confucianism prescribes a hierarchical family structure in which children are expected to demonstrate filial piety (xiao), a deep sense of duty and reverence toward parents and ancestors (Ho, 1994). Within this framework, children’s individual needs, particularly autonomy, are often subordinated to collective family goals and parental authority (Chao, 1994). Taoism further reinforces this orientation by emphasizing harmony and restraint, which discourages children from openly expressing discontent or challenging parental decisions (Chang et al., 2011).

Psychological control may be used by Chinese parents to accomplish the traditional goals set forth by Confucianism and Taosim. A good example is the “shaming” practice—when children fail to meet their expectations, Chinese parents tend to blame them for losing the parents’ “face” and tarnishing the family reputation (Shek, 2008). Rooted in the Confucian concept of *mianzi* (face), this practice ties a child’s personal failures to the social standing of the entire family, which may generate feelings of shame and inadequacy (Fung, 1999). Because the traditional cultural context normalizes such practices as legitimate expressions of parental authority, it is possible that adolescents may not feel uniquely vulnerable; however, on the other hand, given the normalized practice, children may have fewer psychological or societal resources to resist the negative effects of these controlling behaviors. Thus, this controlling practice may diminish adolescents’ self-worth and increase their risk of developing depressive symptoms. Based on IPARTheory and ecological perspectives, we expect that Chinese adolescents will report greater depressive symptoms as they perceive greater parental psychological control. It is important to note that not all families in China strongly endorse traditional hierarchical structures and cultural values; and that as with all cultures, variability in parenting exists depending on their adherence to traditional values, but also other contextual factors such as socioeconomic status, geographic regions, and contemporary social change.

While previous research has established a robust link between parental psychological control and adolescent outcomes, the role of adolescent gender in this link is not well understood. In Chinese families, parents may apply more psychological control to sons due to the traditional favoritism of a male heir over a female one and thus have higher expectations for sons in the Chinese culture to promote the families’ legacy (Tam, 2009). However, daughters may be more vulnerable to parental psychological control as they tend to be more sensitive to parents’ psychological manipulations (C. Fu & Wang, 2021). Thus, we hypothesize that while adolescent sons will report receiving greater parental psychological control, the positive association between parental psychological control and adolescent depressive symptoms will be stronger in daughters.

It is also important to consider the gender of the parent when examining the association between parental psychological control and adolescent well-being (e.g., Bronfenbrenner, 1986). Traditionally, fathers hold the highest status in a Chinese family and are the primary decision-makers in major family affairs yet are less directly involved in daily life events such as homework. In contrast, mothers are the main caregivers who are tasked with day-to-day care of adolescents (Liu et al., 2022) and tending to their emotional needs (Liong, 2017). Many Chinese mothers exhibit the so-called “Tiger Mother” rearing style—mothers who are both highly nurturing and highly intrusive and controlling, especially when it comes to the educational development, decisions, and success of their children (see the literature on “Tiger Mothers” by Chao, 1994; Xie & Li, 2017). These mothers have high hopes for their children and believe it is their responsibility to ensure their academic success. Because of these factors, we believe that Chinese mothers in comparison to fathers will likely exert more psychological control on their children. Past empirical findings are mixed: while some adolescent samples reported fathers as more psychologically and behaviorally (i.e., expectations for good behavior) controlling than mothers (e.g., Shek, 2008), the opposite pattern has also been found (e.g., Zhang et al., 2019). Because of the culture-specific expectation for Chinese mothers, we hypothesize that mothers will be perceived to use more psychological control over their children.

Lastly, family systems theory posits that the family works as a system and therefore it is important to consider the parenting of both parents in the home (Cox & Paley, 2003). For example, research has found that having at least one warm and accepting parent in the home is associated with adolescent resilience such that the experience with the warm and accepting parent may help the promotion of a positive sense of self, which in turn can buffer the negative influence of the other parent using high levels of psychological control (e.g., Rohner, 2021; Van Bronkhorst et al., 2024). On the other hand, adolescents who experience high levels of psychological control by both parents simultaneously are unable to receive any parental messages of acceptance that are needed to develop a positive view of oneself or buffer their experiences with parental psychological control (Heard, 2007; Rogers et al., 2003; Salaam, 2024; Van Bronkhorst et al., 2024; Van Lissa et al., 2019). Thus, we expect a significant interaction effect between mothers’ and fathers’ psychological control on adolescents’ depressive symptoms. In particular, we expect that simultaneous use of psychological control from both parents will amplify adolescents’ depressive symptoms.

Consideration of unique gender dynamics within the Chinese family will provide a more nuanced understanding of the association between parental psychological control and adolescent adjustment (Van Petegem et al., 2022). Traditionally, sons are more highly regarded than daughters, and fathers have absolute authority in the family, thereby creating a noticeable power dynamic based on gender. Past studies have found that higher levels of depression in women compared to males can be explained in part by power gaps between genders in society (Maji, 2018). In addition, the hierarchical family structure sanctioned by Confucianism (Ho, 1994) recognizes fathers as having the greatest authority in the family in part due to their male gender and in part due to their age, because of its association with wisdom. Thus, out of all possible gender dyads in the family, the father–daughter dyad would have the largest gender power gap. Consequently, we hypothesize that in Chinese families, there will be a stronger, positive association between fathers’ use of psychological control and daughters’ depressive symptoms compared to other gender dyads in a family. As no known studies to date have examined the effects of parental psychological control and adolescent depressive symptoms across gender dyads in Chinese families (mother–son, father–son, mother–daughter, father–daughter), the current study addresses this gap in the literature by exploring these associations.

### Current Study

While past research has examined the link between parental psychological control and adolescent well-being in China, the roles of parent and child gender in this relationship are unclear because most of these studies either have combined psychological control across two parents or only examined one parent’s use of control, typically mothers. The current study was designed to address these limitations and to explore both the independent and joint effects of parent gender and adolescent gender in the association between perceived parental psychological control and depressive symptoms in a large sample of Chinese adolescents. Based on cultural ecological and IPAR frameworks as well as previous empirical findings, we hypothesize:

**H_1_.**  *Sons will report greater parental psychological control than daughters.*

**H_2_.**  *Mothers will be perceived to exert greater parental psychological control than fathers.*

**H_3_.**  *The positive association between parental psychological control and depressive symptoms will be stronger for daughters than sons.*

**H_4_.**  *The joint effect of high mother’s and high father’s psychological control will be associated with lower depressive symptoms for adolescent children.*

## 2. Method

### 2.1. Participants and Procedure

Participants were recruited from local middle and high schools in three locations in southern China from 2015 to 2016. The choice of participating schools and classes as well as sample size was based on convenience and not on random sampling. From the initial sample of 3257 Chinese adolescents, only those who reported on both mothers’ and fathers’ psychological control were included in this study. This resulted in a sample of 3069 adolescents (*M_age_* = 15.26, *SD* = 1.67; 45.6% boys and 54.4% girls) from middle and high schools in southern China. A precise response rate was not available; however, according to staff’s recollection, the response rate was high (in the 90% range). Schools that combine middle and high schools are commonly found in China. Adolescents were recruited from three geographic locations in China, including Guangzhou (*n* = 1460), Dongguan (*n* = 400), and Hengdong (*n* = 1209). Of those living in two-parent families (*n* = 79.9%), 77.9% reported living with biological parents.

All procedures performed in this study were approved in line with the ethical standards of the Institutional Review Board of the Guangzhou Medical University in China; and are in accordance with the ethical standards presented in the 1964 Declaration of Helsinki, as well as its later amendments. Informed consent was collected from participants’ guardians and passive assent was collected from participants. Members of the research team distributed paper questionnaires at a convenient time during a class meeting in participating classes. Students were informed of the general purpose of the study and that the study was anonymous and voluntary. They spent approximately 15–20 min completing the questionnaire. No incentive was provided.

### 2.2. Measures

All measures were translated from English to Mandarin by two of the researchers who are bilingual and have PhDs in Psychology. The two authors actively considered culturally sensitive language while adhering to authenticity in translation. When discrepancies arose, the authors debated and negotiated until an agreement was reached. A high school Mandarin teacher proofread the translated version to make sure the language was appropriate for adolescents. Finally, a small pilot study was conducted in Hengdong to ensure that high school students were able to comprehend the questions.

#### 2.2.1. Demographic Questionnaire

Participants reported demographic characteristics such as their age, gender (boy = 0, girl = 1), location (urban = 1, rural = 0), marital status of parents (married = 1, others = 0), sibling status (yes = 1 and no = 0), and parent education level (response choice ranged from 1 = no schooling completed to 13 = doctorate degree).

#### 2.2.2. Parental Psychological Control

Participants completed a 16-item scale that combined items from the *Parental Behavior Inventory and Psychological Control Scale* (Barber, 1996) and self-reported on both their mother’s and father’s use of parental psychological control. A sample item was “(My father/mother) tells me of all the things she/he had done for me.” While this measure has not been formally validated in China, previous research using items from this measure showed good construct validity and internal consistency reliability based on Chinese samples (e.g., Luebbe et al., 2018; Wang et al., 2007). Responses ranged from 1 = *Definitely not like him/her* to 3 = *Definitely like him/her*. Scores were calculated as the mean of 16 items for mothers and for fathers such that higher scores reflected greater perceived psychological control (*α_mother_ =* 0.90 and *α_father_ =* 0.91).

#### 2.2.3. Depressive Symptoms

The 20-item Center for Epidemiologic Studies Depression Scale-Revised (CESD-R) assessed the depressive symptoms of the participants (Eaton et al., 2004). Adolescents rated the frequency of each item (e.g., “I had trouble keeping my mind on what I was doing”) over the past week using a 5-point scale. A sum score was calculated across items. Higher scores indicated more depressive symptoms during the past week (*α* = 0.94).

## 3. Results

Measurement invariance analyses were conducted across boys and girls for measures of depressive symptoms, mother psychological control, and father psychological control. The results indicated a good model fit and supported the comparability of these measures between boys and girls (see Appendix A). Bivariate correlations, skewness, and kurtosis for all study variables are presented for descriptive purposes in Table 1. A parent gender X adolescent gender MANOVA revealed two significant main effects and a significant interaction. In support of H_1_, the main effect of adolescent gender suggested that boys perceived higher levels of parental psychological control than girls [*F*(1, 2996) *=* 25.94, *p* < 0.001; *η*^2^ = 0.01]. In support of H_2_, the main effect of parent gender suggested that adolescents perceived mothers to use more psychological control than fathers [*F*(1, 2996) = 324.45, *p* < 0.001; *η*^2^ = 0.10]. The interaction between parent and adolescent gender [*F*(1, 2996) *=* 36.53, *p* < 0.001; *η*^2^ = 0.01] indicated that boys reported receiving higher father psychological control than girls [*F*(1, 2996) *=* 54.42, *p* < 0.001; *η*^2^ = 0.02], while no significant differences were found between boys’ and girls’ reports of mother psychological control [*F*(1, 2996) *=* 2.97, *p* = 0.09; *η*^2^ = 0.001]. It is important to note that some of the statistically significant effects were small in magnitude (e.g., *η*^2^ = 0.01 and *η*^2^ = 0.001) and should therefore be interpreted with caution. Additionally, an independent sample t-test revealed that girls reported more severe depressive symptoms than boys [*t* (2974) = −5.26, *p* < 0.001; *d* = 0.20]. Correlational analyses indicated that both mothers’ and fathers’ psychological control were significantly and positively related to boys’ and girls’ depressive symptoms (see Table 1 and Table 2).

To explore our research questions on the effect of parent gender X adolescent gender on adolescent depressive symptoms, we adopted the hierarchical regression approach with multiple imputations to address missing data (about 2.71%). As suggested by Enders (2013), 10 imputations were generated to replace missing data using key study variables (gender, location, marital status, sibling status, parental education level, parental psychological control, and depressive symptoms). A five-step hierarchical regression was conducted for the original dataset and repeated for each of the 10 imputations: adolescent depressive symptoms were the dependent variable, mothers’ and fathers’ psychological control and adolescent gender were the predictors. All subsequent analyses were conducted across the 10 imputed datasets, and parameter estimates were pooled using Rubin’s rules. Multicollinearity diagnostics suggested that mothers’ and fathers’ psychological control did not exhibit severe collinearity. Although both variables showed shared variance on the same dimension, the corresponding condition index (13.80) was below conventional thresholds for serious concern. Inspection of the residual plots suggested that the assumptions of linearity and homoscedasticity were generally acceptable, although minor deviations were observed.

Control variables included six key demographic variables: mother and father education, single child or not [single (coded 1) and others (coded 0)], medium-size city (coded 1) or not (coded 0), large city (coded 1) or not (coded 0), and school grade level. All two-way and three-way interaction effects were tested. Prior to entering the regression, all continuous predictors and control variables were standardized. Interaction terms were formed by multiplying the standardized scores of the variables involved in the specific interaction. The five steps of the regression included: In Step 1, control variables were entered, including fathers’ and mothers’ education levels, adolescent only-child status, and location (urban vs. rural). In Step 2, the main predictors—father’s psychological control and mother’s psychological control—were entered. In Step 3, adolescent gender was entered. In Step 4, all two-way interaction terms were entered: mother control * gender, father control * gender, and mother control * father control. Finally, in Step 5, the three-way interaction term (mother control * father control * gender) was entered. Pooled results across the original dataset and the 10 imputations (e.g., unstandardized regression coefficients) were generated and reported below. This analysis was conducted in SPSS version 30 (IBM Corporation, Armonk, NY; see Table 1 and Table 2).

The results revealed that the three-way interaction between mother’s psychological control, father’s psychological control, and adolescent’s gender was not significant (*B* = 0.78, *p* = 0.10). Therefore, the results of significant two-way interactions from Step 4 were reported (see Table 3). For each significant two-way interaction, simple slope analyses were conducted to probe the nature of the interaction, and the results of these tests are presented alongside the regression findings.

**Figure 1:**
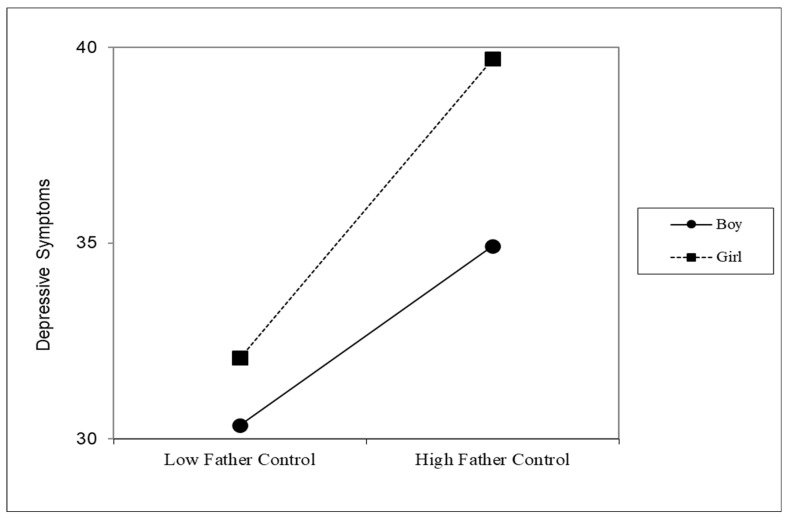
Estimated level of depression by father psychological control, stratified by gender.

The results from the regression analyses indicated that both mother’s (*B* = 3.72, *p* < 0.001) and father’s (*B* = 1.37, *p* = 0.01) psychological control were positively associated with adolescent depressive symptoms (see Table 2). It also revealed a significant two-way interaction between mother’s vs. father’s control and adolescent gender (*B* = 2.20, *p* < 0.01). Further probing of the interaction showed that the positive association between father’s psychological control and depressive symptoms was stronger for daughters (*B* = 3.82, *p* < 0.01) than sons (*B* = 2.29, *p* < 0.01; see Table 3 and Figure 1). No significant two-way interaction was found between mother’s psychological control and adolescent’s gender on depressive symptoms (*B* = −0.15, *p* = 0.83).

**Figure 2:**
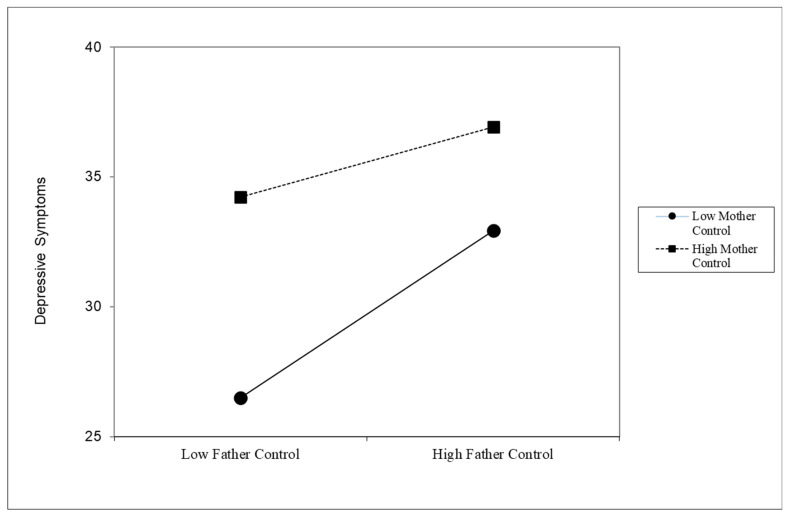
Estimated level of depressive symptoms by father’s psychological control, stratified by mother’s psychological control.

Lastly, the joint effect between father and mother control resulted in a significant two-way interaction (*B* = −0.81, *p* < 0.01). The simple slope test revealed a significant negative coefficient indicating that the incremental association of father psychological control and adolescent depressive symptoms weakened when mother psychological control was high (*B* = 1.35, *p* = 0.02) compared to when mother psychological control was low (*B =* 3.22, *p* < 0.01) (see Table 3 and Figure 2). See Appendix B for results from the full hierarchical regression model.

## 4. Discussion

Although Chinese parents’ use of psychological control has been linked with adolescent mental health (Bi et al., 2024), no studies to our knowledge have considered how the association may differ across gender dyads of parents and adolescents and minimal research has examined the joint influences of Chinese mothers’ and fathers’ use of psychological control on adolescent depressive symptoms. Guided by sociocultural and familial frameworks, findings from this study provide a more nuanced understanding of how parent and adolescent gender may influence the link between parental psychological control and adolescent depressive symptoms, and thus inform the research, programs, and practice of mental health professionals who work with Chinese adolescents and their families.

Our study revealed that more adolescent depressive symptoms were associated with greater perceptions of mothers’ and fathers’ use of parental psychological control. These findings are consistent with previous studies that have also demonstrated a positive association between parental psychological control and Chinese adolescent depressive symptoms (Cui et al., 2014; Nigela et al., 2015). Providing further support for IPARTheory, the current findings suggest that regardless of the specific culture, parents’ intrusive and rejecting behaviors such as psychological control may adversely affect adolescent self-determination, identity development, and self-esteem, rendering them to engage in more self-criticism and to experience greater depressive symptoms (Rohner, 2021).

Taking the MANOVA and regression results together, an intriguing pattern emerged: even though daughters reported fathers’ use of psychological control to be lower than sons did, the positive association between daughters’ depressive symptoms and their perceptions of fathers’ use of psychological control was significantly stronger compared to sons. This finding supports past empirical findings revealing gender differences in adolescent perceptions of negative paternal parenting practices, where daughters were more negatively impacted by negative interpersonal interactions and unwanted parenting practices than sons (Zhu & Shek, 2021). Additionally, it aligns with two meta-analyses indicating that, in traditional societies, adults’ recollections of fathers’ use of psychological control were more strongly and positively associated with psychological maladjustment in adult daughters than in adult sons (Ali et al., 2015; Rohner, 2021). Thus, findings from this study are a major extension of past studies on parent–adolescent relationships, particularly father–daughter relationships (X. Fu et al., 2021), because they clearly indicate that while parental psychological control may have a consistent, negative association with adolescent depressive symptoms, the amount of control may not be the most critical factor in determining the impact of parental psychological control. Instead, we suspect the very large power discrepancy based on gender between fathers and their daughters in Chinese families may be an important yet understudied reason for this intriguing pattern. Indeed, the traditional hierarchical family structure endorsed by Confucianism entitles greater authority to fathers due to the combination of male gender and seniority (Ho, 1994). Past research has found that women are more likely to experience depressive symptoms than men in part because of gendered power dynamics (Maji, 2018). Thus, the father–daughter dyad may be the most vulnerable gender dyad in Chinese families to experiencing a positive association between parental psychological control and depressive symptoms. Additional studies are needed to explore the specific mechanism through which a father’s psychological control is associated with their daughter’s well-being, particularly over time.

Furthermore, results from the current study support the family system theory by demonstrating the importance of considering the parenting of both mothers and fathers in the family. Figure 2 shows that a father’s use of greater psychological control was only significantly associated with an adolescent’s depressive symptoms when a mother’s psychological control was low. In other words, the association between fathers’ control and children’s depressive symptoms weakened when mothers’ control was high. These findings suggest that while both parents’ psychological control tended to have a negative association with an adolescent’s mental health, mothers’ control appears to carry greater weight in this relationship. Although Chinese mothers may not traditionally be considered the primary authority figures within the family, they hold a vital role as nurturers and supporters of their children’s current well-being due to the mother’s main caregiver role (Chao, 1994). It is important to note that due to the cross-sectional nature of this study, we cannot rule out the possibility that when adolescents demonstrated greater depressive symptoms, it may have caused fathers to exert more psychological control at home. Previous research has indicated such a bidirectional pathway: Cao and Liu (2023) found that the quality of the Chinese parent–child relationship predicted adolescents’ emotional traits, and that an adolescent’s own emotional characteristics predicted subsequent changes in the parent–child relationship quality.

A major strength of this study is its consideration of the associations between parental psychological control and adolescent depressive symptoms across specific parent–adolescent gender dyads within Chinese families (i.e., mother–son, mother–daughter, father–son, father–daughter). This dyadic approach offers a more nuanced understanding of how gender dynamics may shape the impact of parenting practices on adolescents’ mental health. Another important contribution of the current study is its examination of the joint effects of both mothers’ and fathers’ use of psychological control. By simultaneously considering the roles of both parents, the study acknowledges the interdependent nature of parenting in Chinese families, where maternal and paternal influences may interact to shape adolescent outcomes in unique and nuanced ways.

There are several noteworthy limitations to discuss. The current study is a cross-sectional study that limits the ability to explore causal explanations. Longitudinal research on adolescent–parent gender dyads would be extremely helpful to understand the developmental trajectory and causal direction of the parental psychological control–adolescent depression link. A longitudinal design would have also allowed us to account for the nested nature of collecting surveys from middle and senior high schools in three different geographic regions in China. Also, the study measures were based entirely on adolescent self-reports, which may threaten the internal validity of the findings due to shared method variance and response biases. Even though adolescents’ perceptions of parental psychological control are important for understanding other internal characteristics such as adolescents’ depressive symptoms, it would be informative if future research measured parental psychological control by using more objective methods such as observations or by including multiple reporters such as parents, peers, and teachers.

To our knowledge, this study is the first to consider gender dyads of parents and adolescents when examining the association between perceived psychological control and depressive symptoms in Chinese families. Given that our findings indicate that Chinese daughters are particularly vulnerable to the negative effects of psychological control by fathers, it is important that mental health programs include fathers in their treatment plans. Future research and preventive interventionists seeking to strengthen adolescent and family well-being must continue to consider sociocultural and gendered norms that intersect and influence parent–adolescent interactions.

## Figures and Tables

**Table 1:** Demographic information of the sample (frequency).

Variable	Category	*n*	%
Gender	Male	1367	44.50%
	Female	1631	53.10%
	Missing	71	2.30%
Only child	Yes	863	28.10%
	No	2186	71.20%
	Missing	20	0.70%
Location	Rural	1209	39.40%
	Mid-sized city	400	13%
	Large city	1460	47.60%
School grade	Middle school	1479	48.20%
	High school	1550	50.50%
	Missing	40	1.30%

**Table 2:** Correlations, means, and standard deviations (SDs) for study variables by gender.

	1	2	3
1. Depression		0.23 **	0.17 **
2. Mother Control	0.30 **		0.63 **
3. Father Control	0.28 **	0.53 **	
M_boy_ (*SD*)	34.64 (16.06)	1.73 (0.47)	1.64 (0.49)
M_girl_ (*SD*)	37.84 (16.14)	1.70 (0.47)	1.51 (0.44)
Skewness_boy_	1.32	0.71	0.91
Skewness_girl_	1.24	0.71	1.15
Kurtosis_boy_	1.93	0.11	0.44
Kurtosis_girl_	1.36	−0.14	1.02

Note. *N* = 3069 (boys = 1403; girls = 1666); correlations below diagonal are for girls and above diagonal are for boys. ** *p* < 0.01.

**Table 3:** Step 4 of the hierarchical regression model for 2-way interactions on adolescents’ depressive symptoms.

Variables	B	Std. Error	t	Sig.
Step 4				
Father Education	0.23	0.18	1.30	0.20
Mother Education	−0.09	0.17	−0.53	0.60
Only Child	−0.89	0.71	−1.25	0.21
Medium City	0.95	0.97	0.98	0.33
Big City	**−1.99**	0.72	−2.74	0.01
School Grade Level	**3.46**	0.61	5.66	<0.001
Mother Control	**3.72**	0.54	6.89	<0.001
Father Control	**1.37**	0.53	2.60	0.01
Gender	**3.51**	0.57	6.12	<0.001
MC*G	−0.15	0.71	−0.21	0.83
FC*G	**2.20**	0.70	3.16	<0.001
MC*FC	**−0.81**	0.22	−3.67	<0.001
*R*^2^ (Step 4)	**0.12**			
*R*^2^Δ (Step 3–Step 4)	**0.01**			

Note. Values are unstandardized Bs. FC*MC = Father Control*Mother Control; MC*G = Mother Control*Gender; FC*G = Father Control*Gender. Bolded values indicate statistically significant coefficients (*p* < 0.05). See Appendix B for results from the full hierarchical regression model.

## Data Availability

The data presented in this study are available on reasonable request from the second author. The data are not publicly available due to confidentiality and research ethics.

## Appendix A. Measurement Invariance Across Boys and Girls

**Measure**	**Model**	**χ^2^**	**df**	**CFI**	**TLI**	**RMSEA**	**SRMR**
MC	Configural	1850.68	200	0.917	0.900	0.074	0.048
	Metric	1894.22	215	0.915	0.905	0.072	0.051
	Scalar	1980.07	230	0.912	0.908	0.071	0.052
FC	Configural	2036.95	200	0.915	0.898	0.078	0.048
	Metric	2076.39	215	0.914	0.904	0.076	0.050
	Scalar	2159.85	230	0.910	0.907	0.075	0.053
DEP	Configural	3357.73	324	0.905	0.889	0.079	0.043
	Metric	3448.12	343	0.903	0.892	0.078	0.050
	Scalar	3551.10	362	0.900	0.895	0.077	0.051
Note. MC = mother control; FC = father control; DEP = depression. As shown in Table 1, MC, FC, and DEP all demonstrated good general equality across boys and girls, with evidence for configural, metric, and near-scalar invariance in the revised models.							

## Appendix B. Full Results for the Hierarchical Regression for 2-Way Interactions on Adolescents’ Depressive Symptoms

**Steps**	**Variables**	***B***	***SE***	***t***	***p***
Step 1	Constant	36.61	1.00	36.59	<0.001
	Father Education	0.21	0.19	1.12	0.27
	Mother Education	−0.19	0.18	−1.03	0.30
	Only Child	−1.00	0.75	−1.33	0.18
	Medium City	0.06	1.02	0.06	0.95
	Big City	−1.97	0.76	−2.59	0.01
	School Grade Level	1.51	0.63	2.38	0.02
Step 2	Constant	34.82	0.93	37.26	<0.001
	Father Education	0.24	0.18	1.36	0.18
	Mother Education	−0.08	0.17	−0.46	0.64
	Only Child	−1.27	0.71	−1.77	0.08
	Medium City	1.11	0.98	1.13	0.26
	Big City	−2.19	0.73	−3.02	<0.001
	School Grade Level	3.33	0.62	5.39	<0.001
	Mother Control	3.66	0.35	10.55	<0.001
	Father Control	1.70	0.35	4.92	<0.001
Step 3	Constant	32.83	0.98	33.66	<0.001
	Father Education	0.23	0.18	1.27	0.21
	Mother Education	−0.08	0.17	−0.48	0.63
	Only Child	−0.87	0.71	−1.22	0.22
	Medium City	0.72	0.97	0.74	0.46
	Big City	−2.02	0.72	−2.79	0.01
	School Grade Level	3.42	0.61	5.58	<0.001
	Mother Control	3.54	0.35	10.25	<0.001
	Father Control	1.98	0.35	5.72	<0.001
	Gender	3.54	0.58	6.15	<0.001
Step 4	Constant	33.43	0.99	33.92	<0.001
	Father Education	0.23	0.18	1.30	0.20
	Mother Education	−0.09	0.17	−0.53	0.60
	Only Child	−0.89	0.71	−1.25	0.21
	Medium City	0.95	0.97	0.98	0.33
	Big City	−1.99	0.72	−2.74	0.01
	School Grade Level	3.46	0.61	5.66	<0.001
	Mother Control	3.72	0.54	6.89	<0.001
	Father Control	1.37	0.53	2.60	0.01
	Gender	3.51	0.57	6.12	<0.001
	MC*G	−0.15	0.71	−0.21	0.83
	FC*G	2.20	0.70	3.16	<0.001
	MC*FC	−0.81	0.22	−3.67	<0.001
Step 5	Constant	33.59	0.99	33.85	<0.001
	Father Education	0.23	0.18	1.30	0.20
	Mother Education	−0.09	0.17	−0.50	0.62
	Only Child	−0.92	0.71	−1.30	0.20
	Medium City	0.95	0.97	0.98	0.33
	Big City	−1.94	0.73	−2.67	0.01
	School Grade Level	3.46	0.61	5.66	<0.001
	Mother Control	3.83	0.54	7.08	<0.001
	Father Control	1.56	0.54	2.89	<0.001
	Gender	3.06	0.62	4.91	<0.001
	MC*G	−0.20	0.70	−0.29	0.77
	FC*G	1.74	0.75	2.32	0.02
	MC*FC	−1.16	0.30	−3.86	<0.001
	MC*FC*G	0.78	0.47	1.66	0.10
Note. B = unstandardized coefficient; SE = standard error; MC = mother psychological control; FC = father psychological control; G = gender. Step 1 included the control variables. Step 2 added MC and FC. Step 3 added G. Step 4 added the two-way interaction terms (MC*G, FC*G, and MC*FC). Step 5 added the three-way interaction term (MC*FC*G). Coefficients are based on pooled estimates from multiple imputations.					
